# Supplementary material for: Oxygen-rich interface enables reversible stibium stripping/plating chemistry in aqueous alkaline batteries
Source: Nat Commun. 2021 Jan 4;12:14. doi: 10.1038/s41467-020-20170-8 (PMC7782749; doi:10.1038/s41467-020-20170-8)
Supplement: Supplementary file 1 — Supplementary Information [file 41467_2020_20170_MOESM1_ESM.pdf]

Supplementary Information

Oxygen-rich interface enables reversible stibium stripping/plating chemistry in aqueous alkaline batteries

Zhang *et al.*

Supplementary Figures

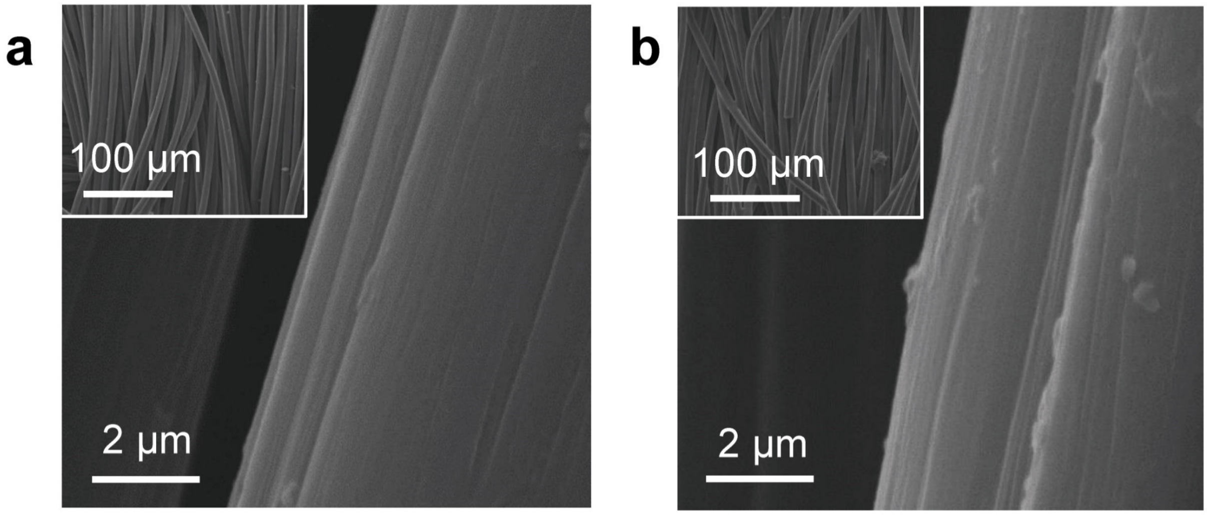

Supplementary Figure 1 Scanning electron microscopy (SEM) images of **a** CS and **b** FCS.

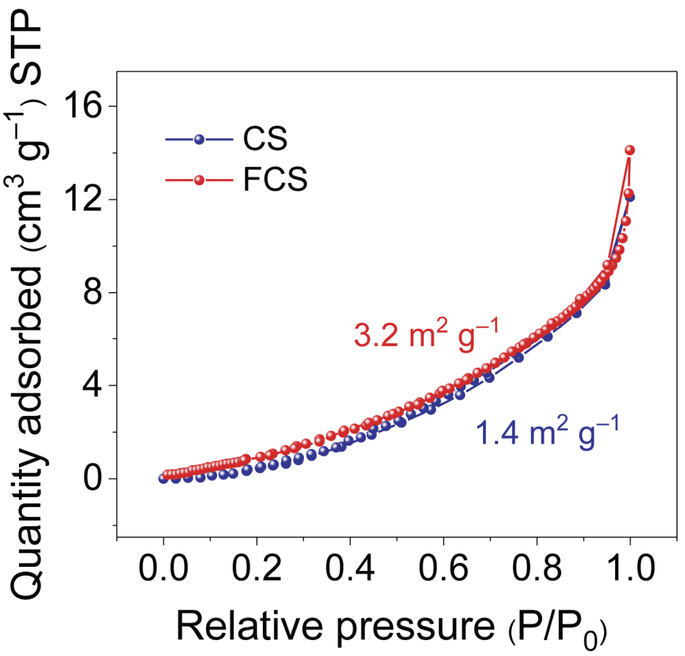

Supplementary Figure 2 N_2 physisorption isotherms at 77 K of the CS and FCS. The Brunauer–Emmett–Teller (BET) surface area of CS and FCS are 1.4 and 3.2 m^2 g^{-1}, respectively.

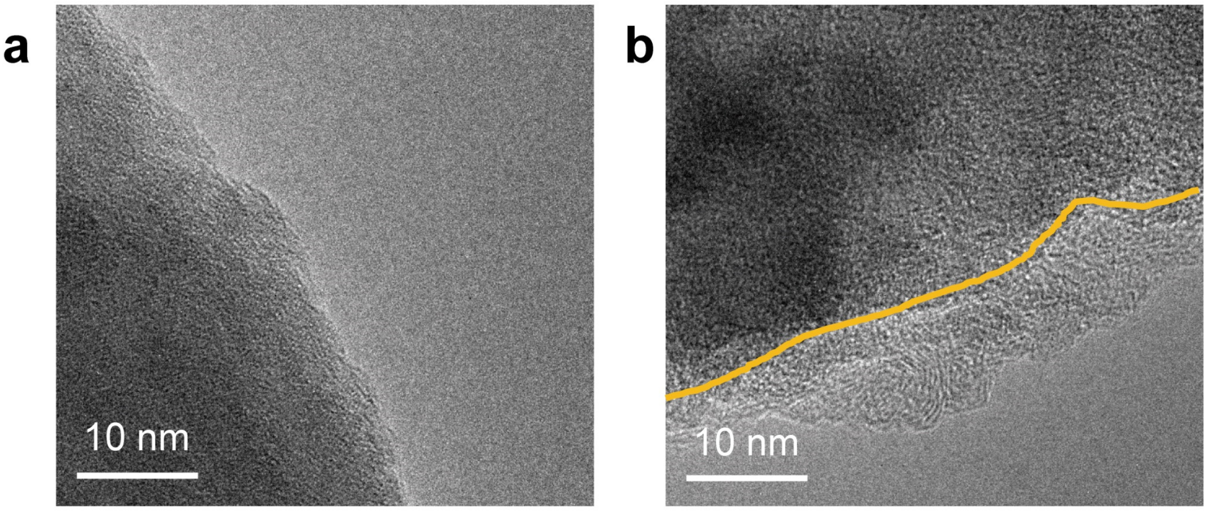

Supplementary Figure 3 High-resolution transmission electron microscopy (HRTEM) images of **a** CS and **b** FCS.

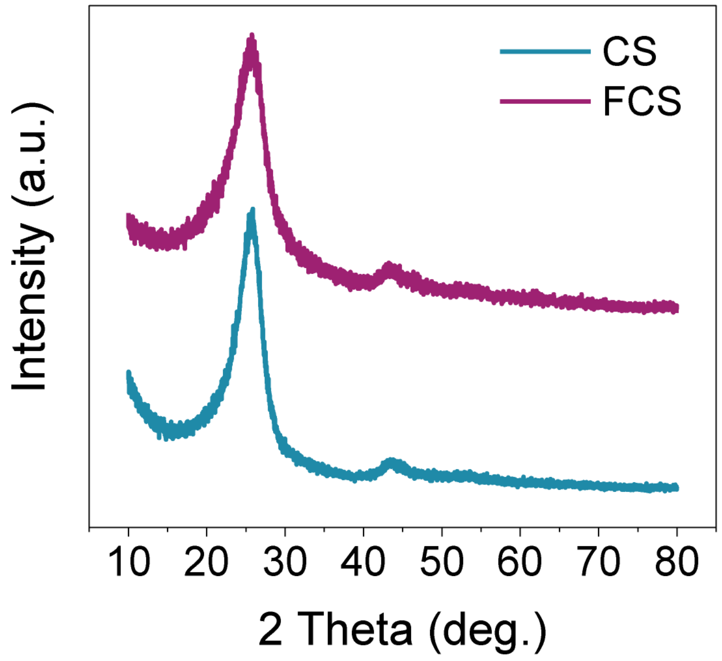

Supplementary Figure 4 X-ray diffraction (XRD) spectra of CS and FCS.

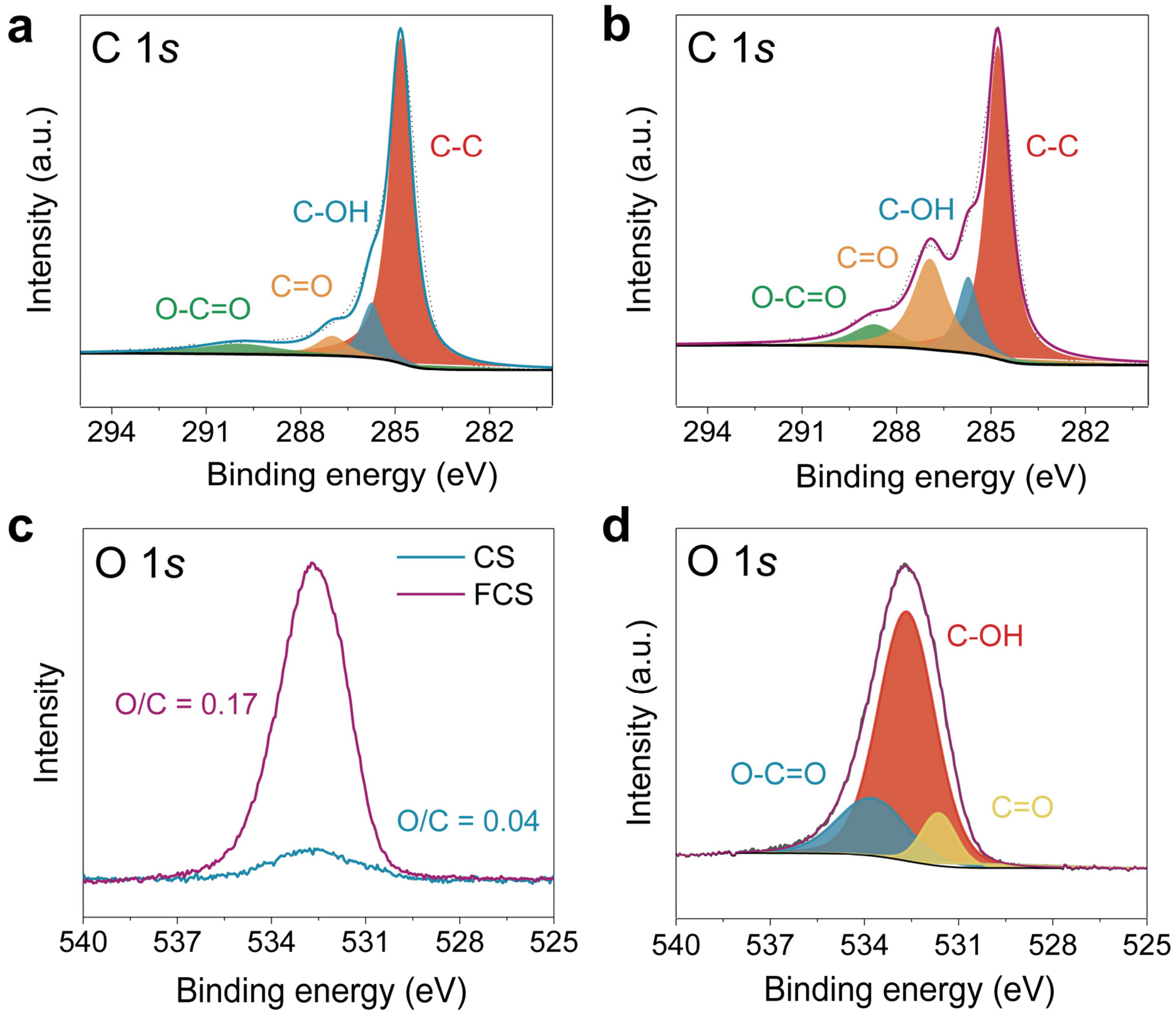

Supplementary Figure 5 The core level C 1*s* X-ray photoelectron spectroscopy (XPS) spectra of **a** CS and **b** FCS. **c** The intensity of the O 1*s* peak of CS and FCS. The O/C ratios of CS and FCS are 0.04 and 0.17, respectively. **d** The core level O 1*s* XPS spectra of FCS.

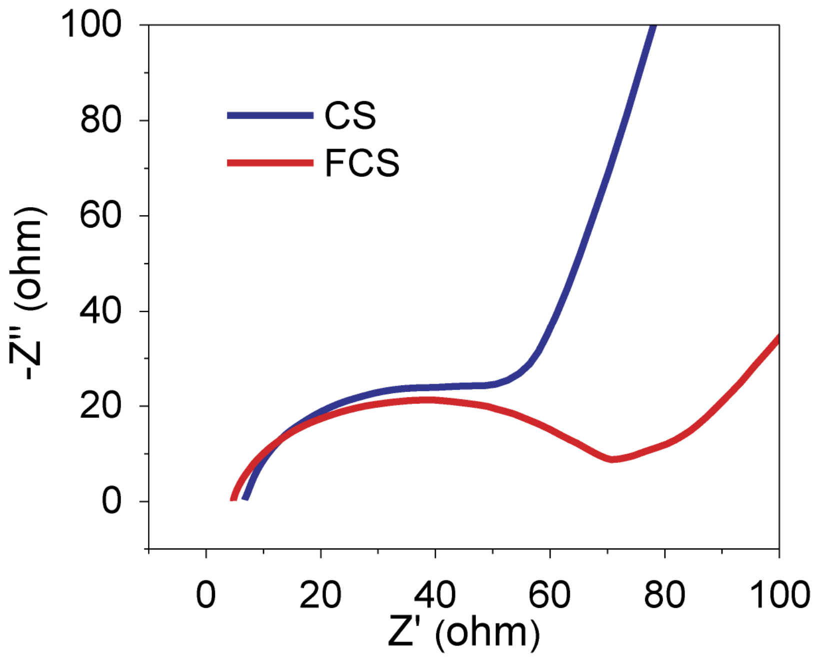

Supplementary Figure 6 Nyquist plots of CS and FCS. The charge transfer resistance of FCS is slightly increased.

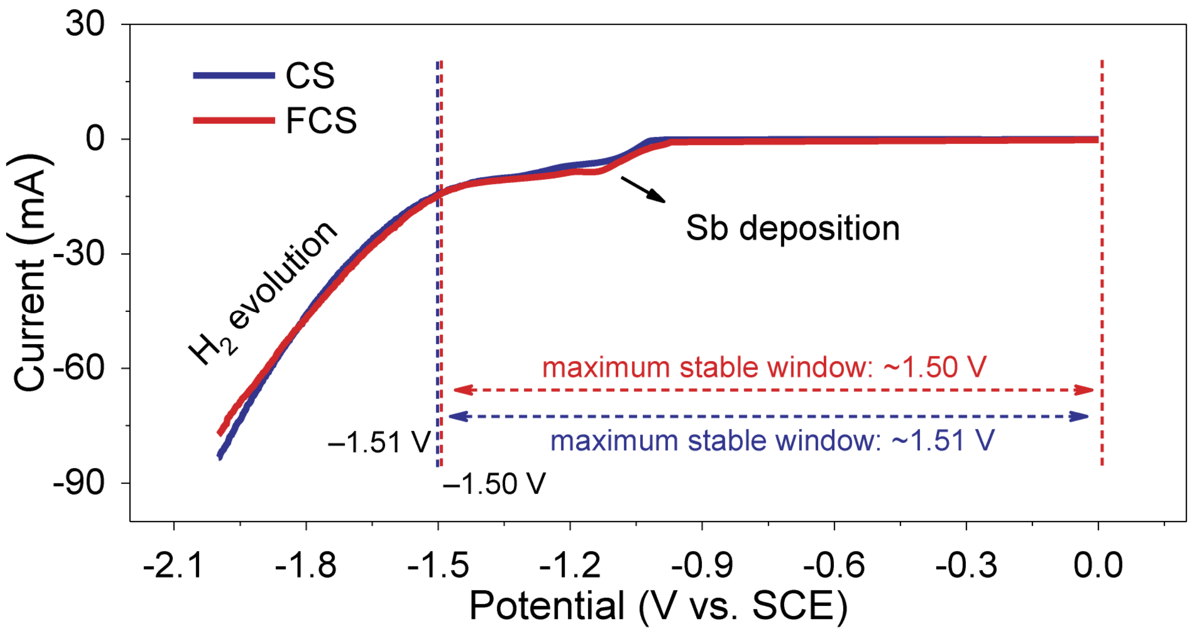

Supplementary Figure 7 Working potential window of the CS and FCS electrodes in 1 M KOH with 0.027 M $C_8H_4K_2O_{12}Sb_2$. The overpotential of hydrogen evolution reaction (HER) on the CS and FCS are ~435 and ~425 mV, respectively.

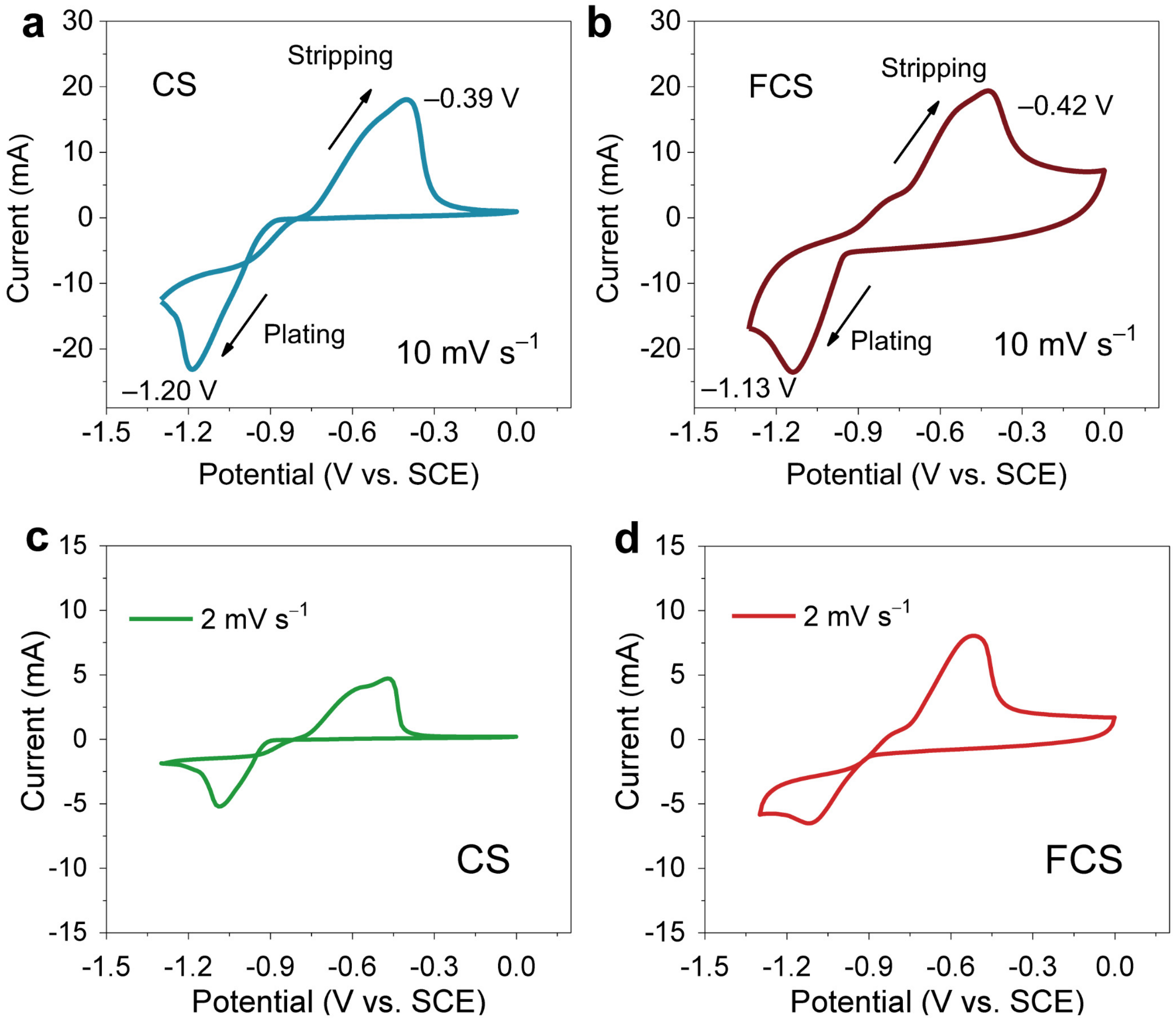

Supplementary Figure 8 The stripping/plating cyclic voltammograms (CV) curves of Sb on **a** CS and **b** FCS at 10 mV s^{-1}; on **c** CS and **d** FCS at 2 mV s^{-1}. FCS shows smaller polarization at each condition.

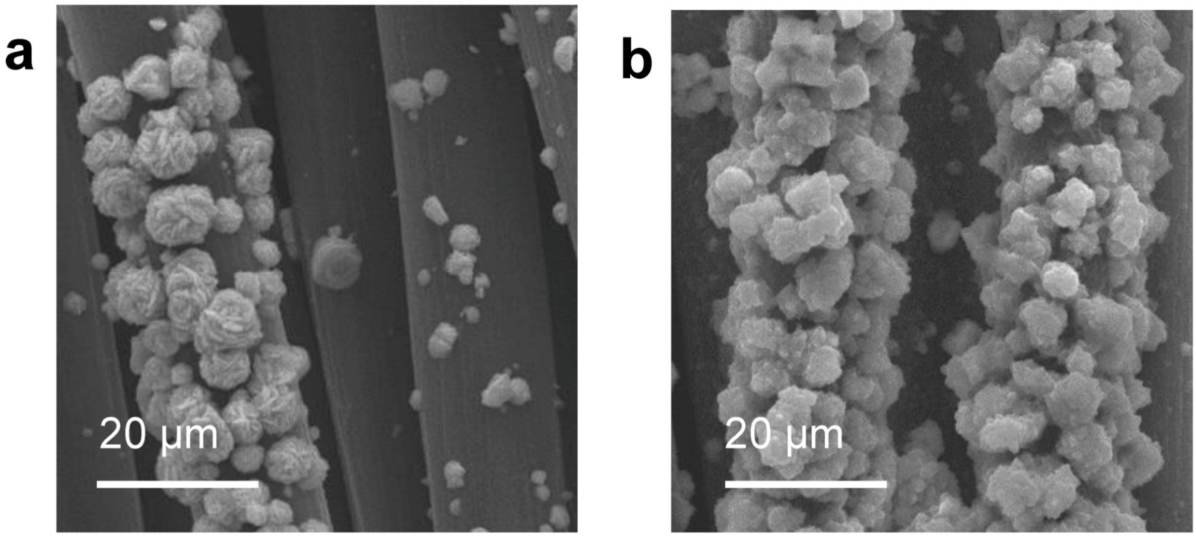

Supplementary Figure 9 Low resolution SEM images of **a** Sb/CS and **b** Sb/FCS.

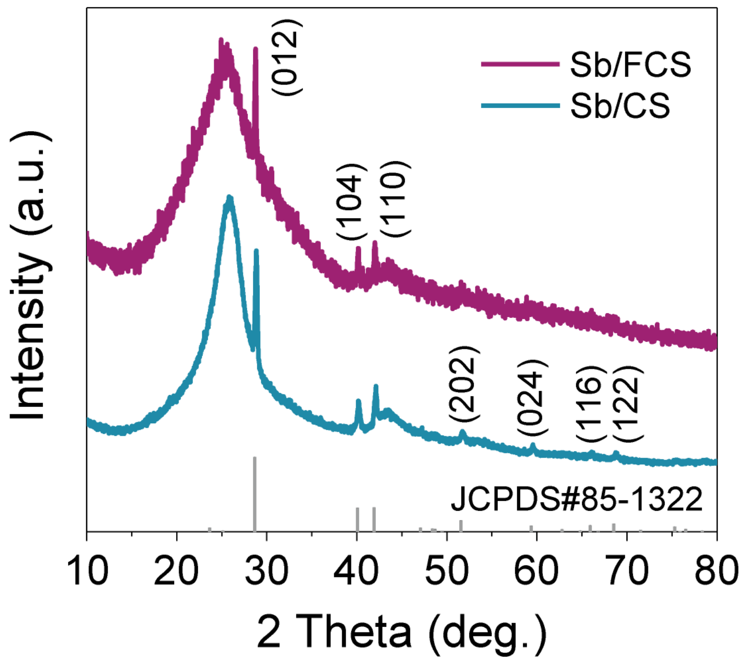

Supplementary Figure 10 XRD spectra of Sb/CS and Sb/FCS.

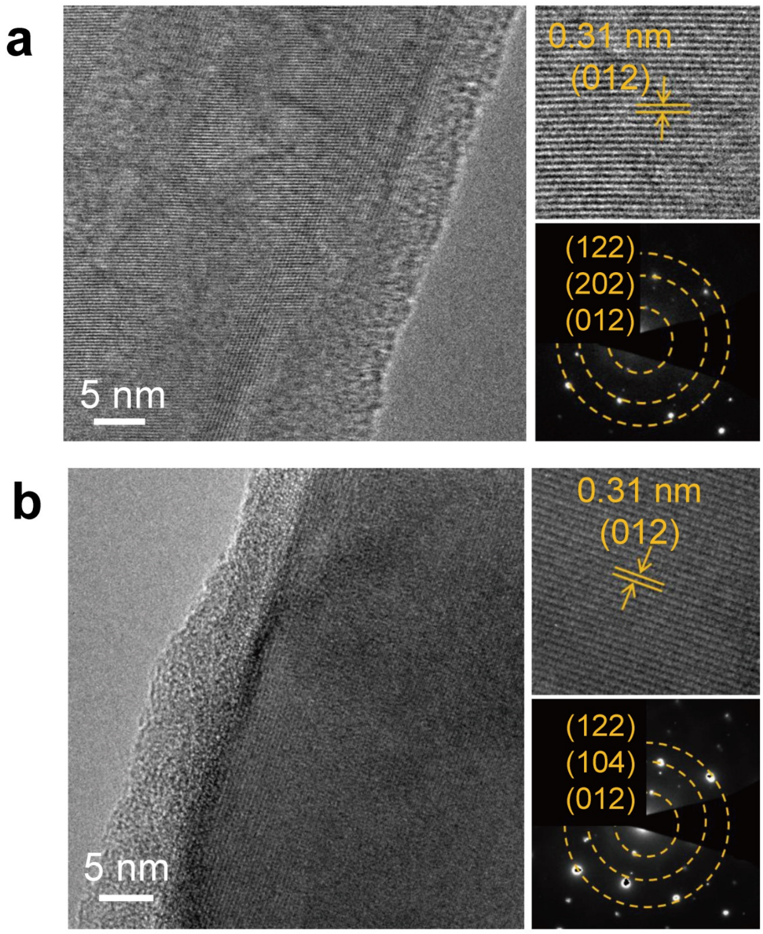

Supplementary Figure 11 HRTEM images and selected-area electron diffraction (SAED) patterns of **a** Sb/CS and **b** Sb/FCS.

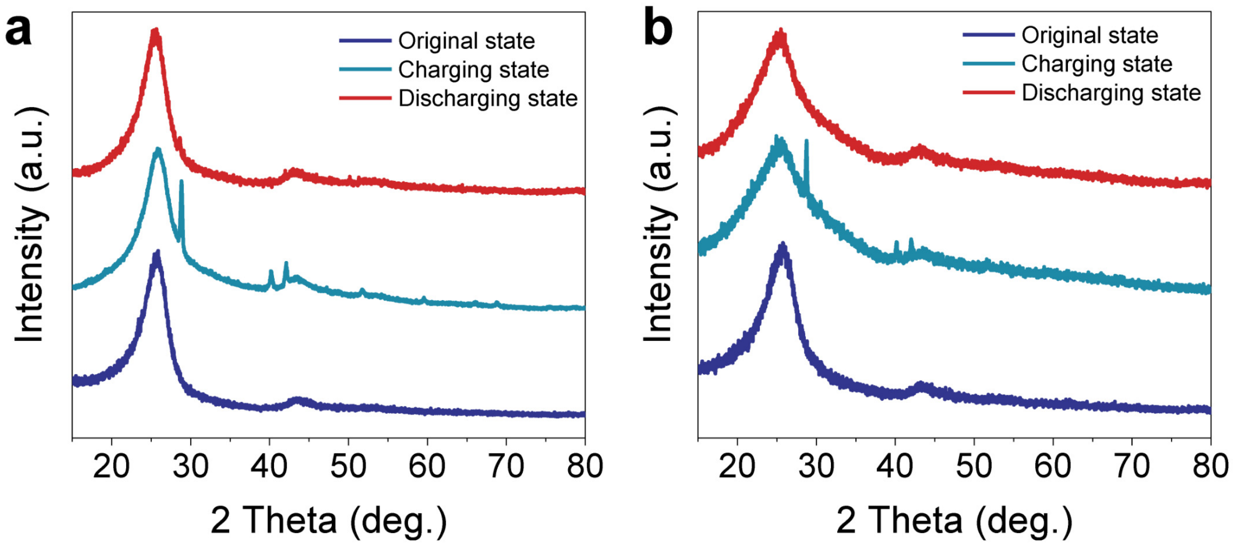

Supplementary Figure 12 XRD spectra of **a** Sb/CS and **b** Sb/FCS in original state, charging state and discharging state.

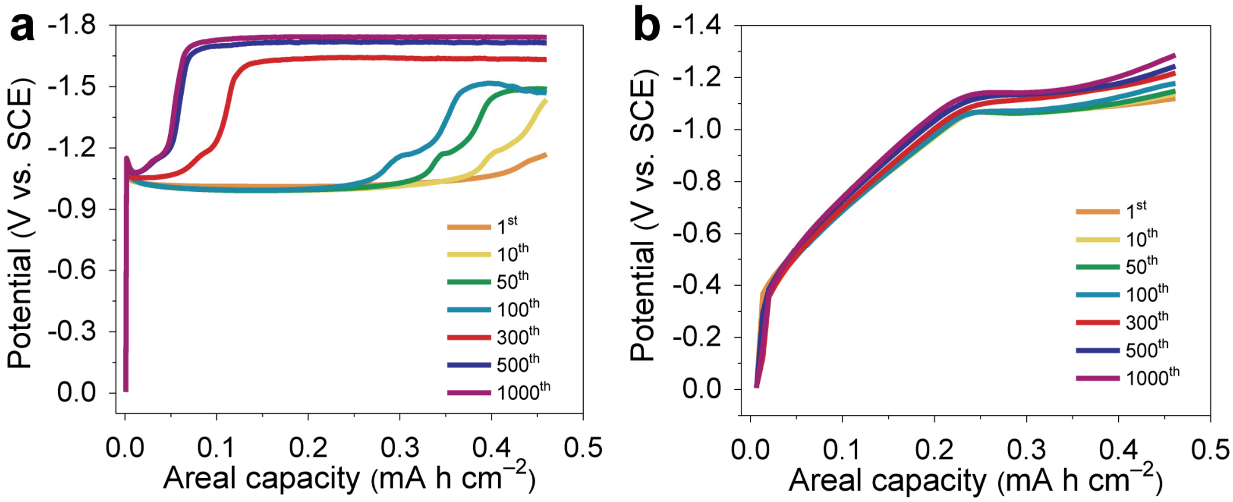

Supplementary Figure 13 Charging curves at different cycling numbers with a fixed charging capacity of 0.47 mA h cm^{-2} at 30 mA cm^{-2} of **a** Sb/CS and **b** Sb/FCS.

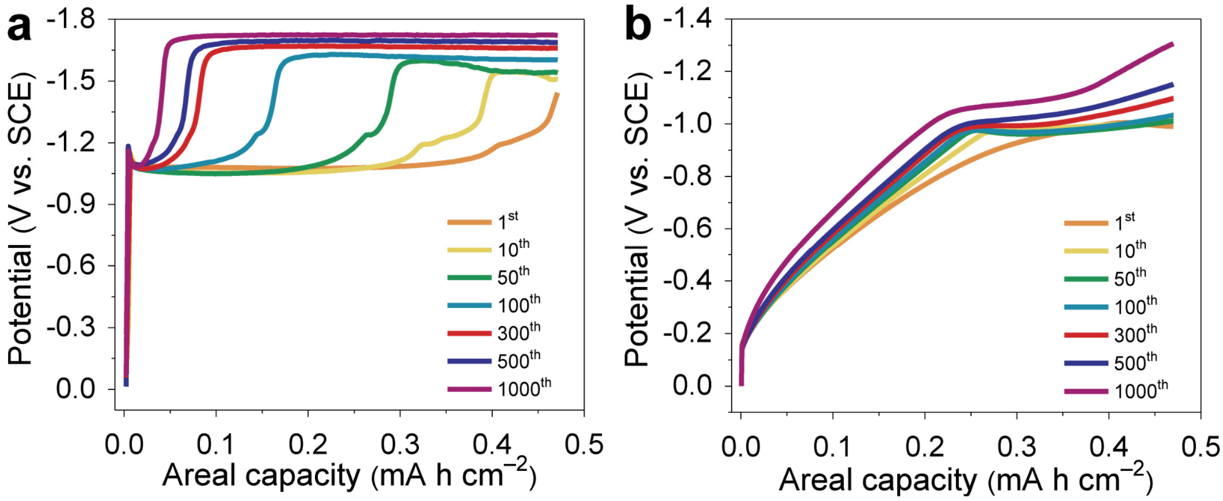

Supplementary Figure 14 Charging curves at different cycling numbers with a fixed charging capacity of 0.47 mA h cm^{-2} at 20 mA cm^{-2} of **a** Sb/CS and **b** Sb/FCS.

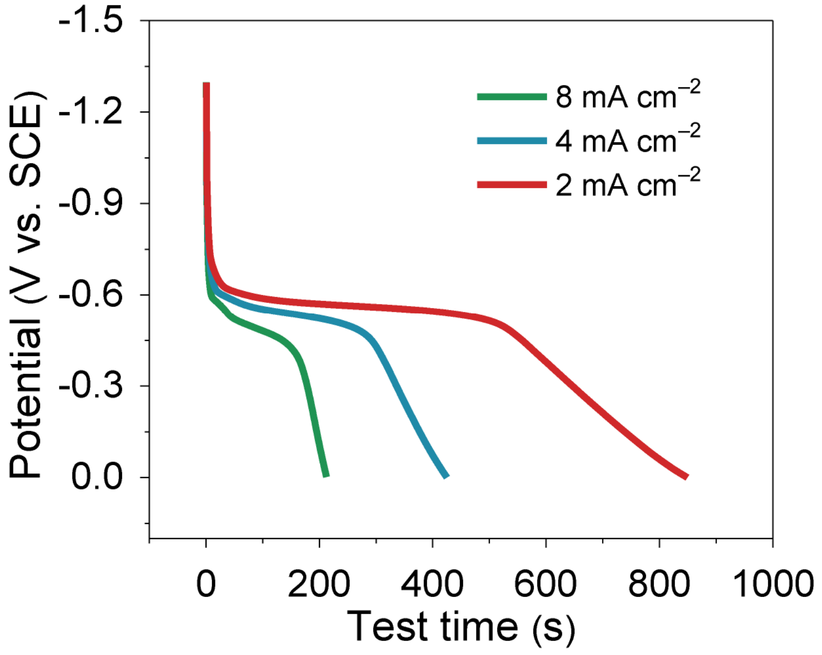

Supplementary Figure 15 Galvanostatic discharging curves at different current densities of Sb/FCS.

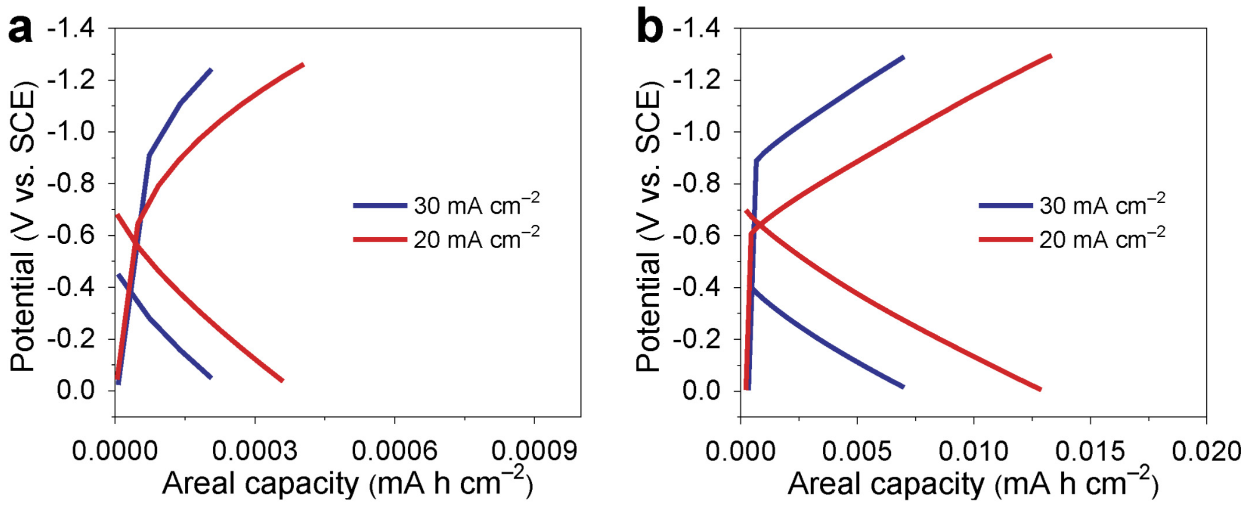

Supplementary Figure 16 Galvanostatic charge-discharge (GCD) curves in 1 M KOH of bare **a** CS and **b** FCS.

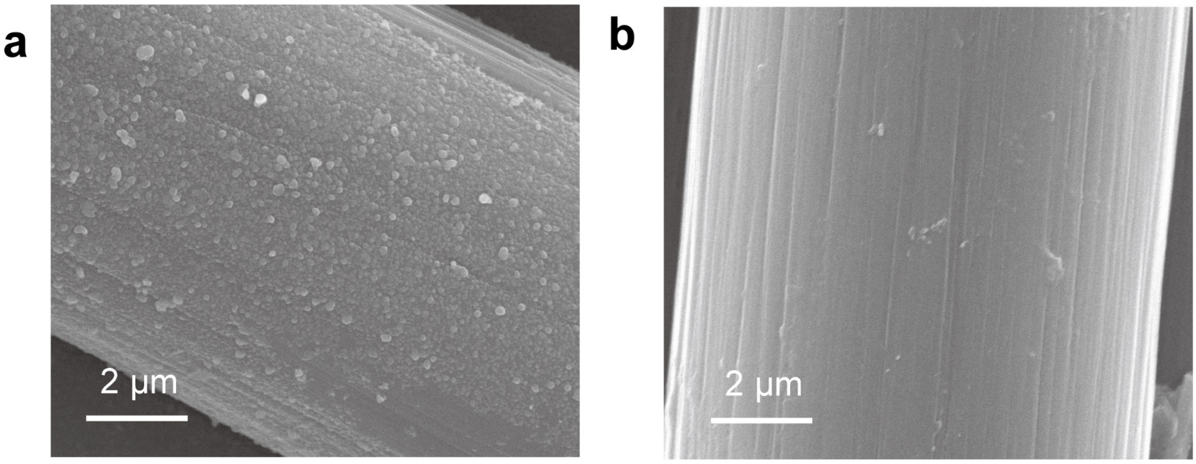

Supplementary Figure 17 SEM images at discharging state after 1000 cycles under 20 mA cm^{-2} of **a** CS and **b** FCS.

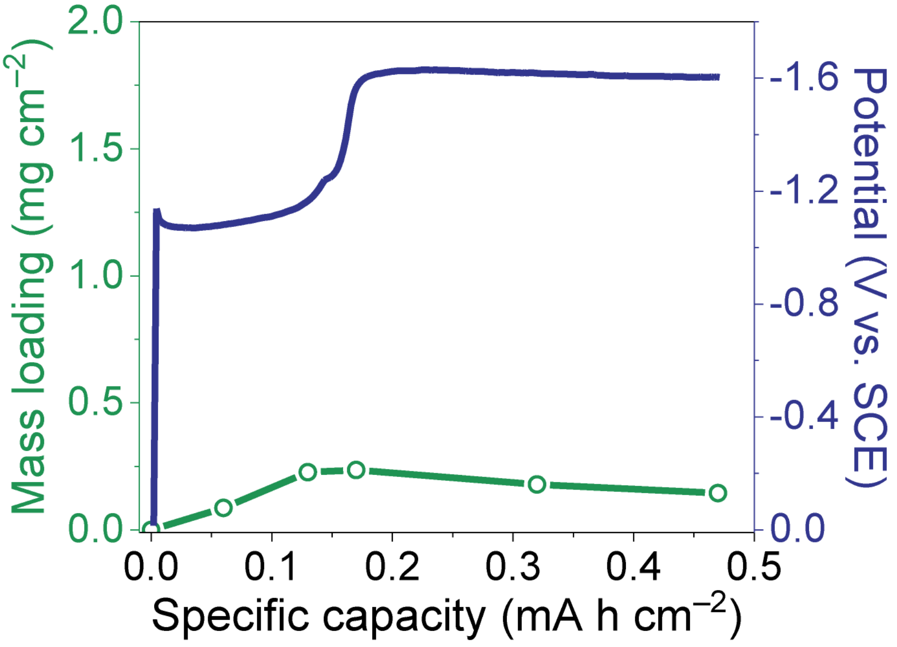

Supplementary Figure 18 Weight variation curve of CS during the Sb deposition process.

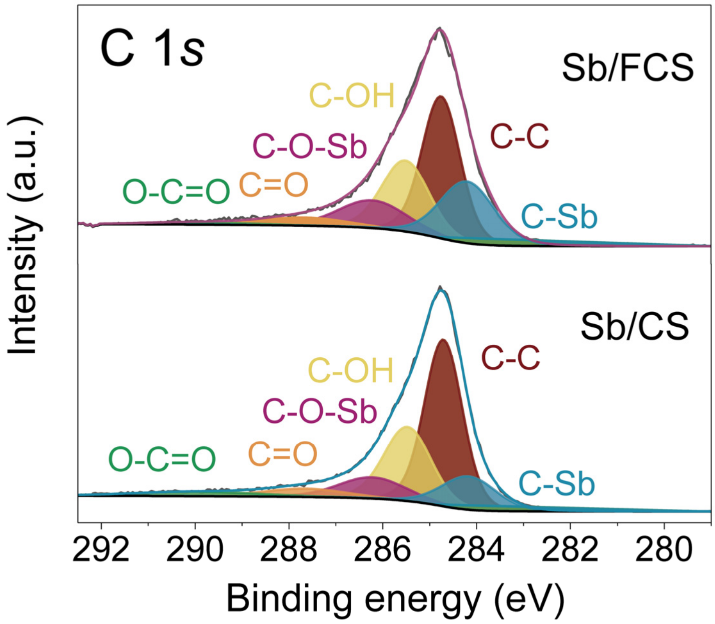

Supplementary Figure 19 The core level C 1*s* XPS spectra of Sb/CS and Sb/FCS.

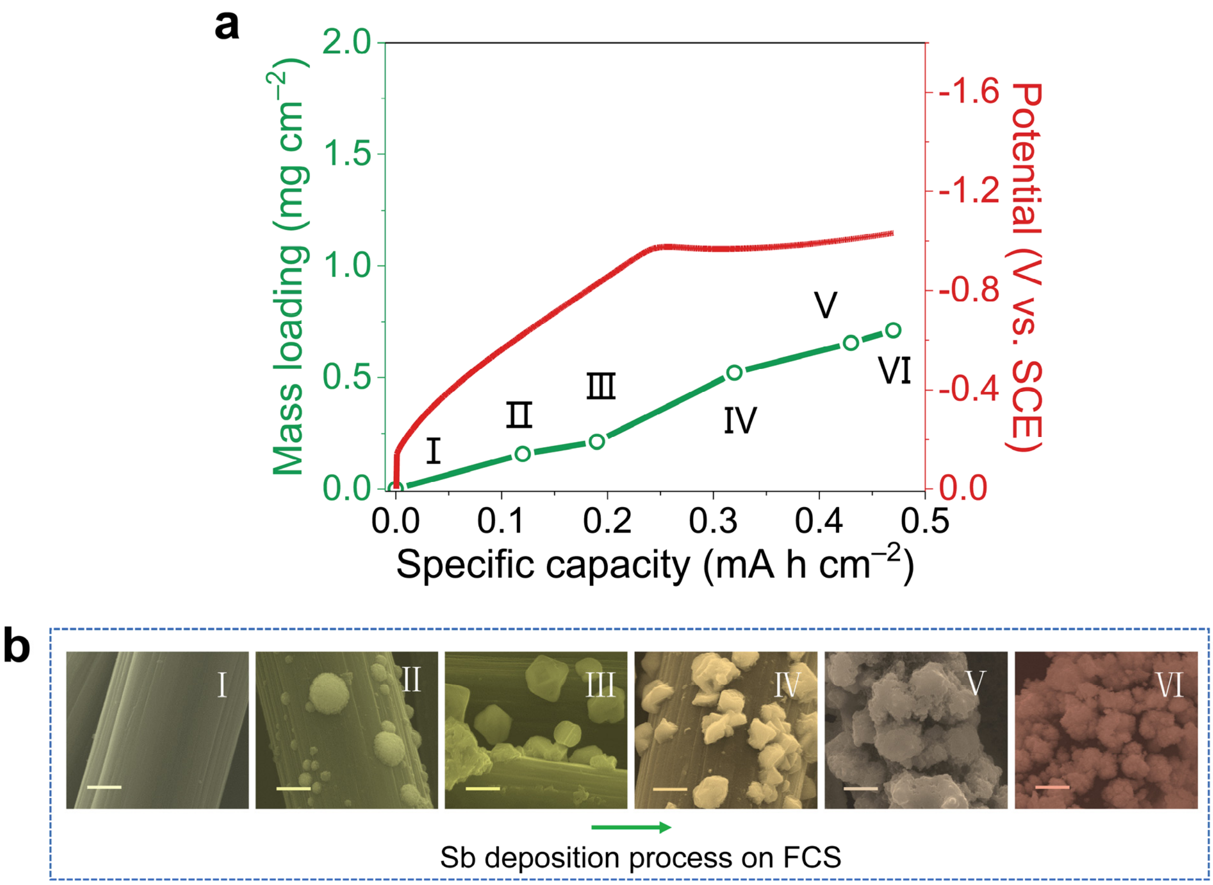

Supplementary Figure 20 a Weight variation curve and **b** ex-situ SEM images of FCS during the Sb deposition process.

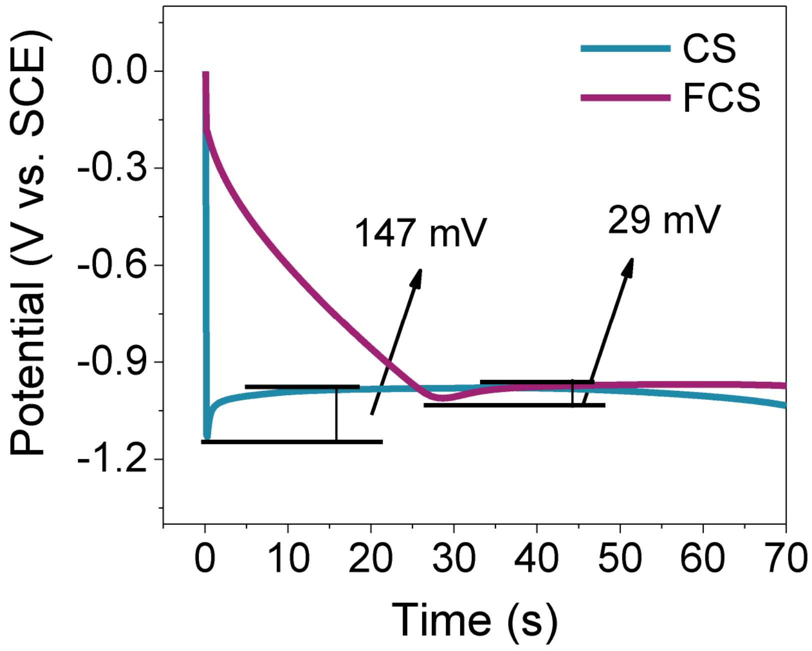

Supplementary Figure 21 The voltage–time curves during Sb nucleation at 20 mA cm^{-2} on bare CS and FCS electrodes.

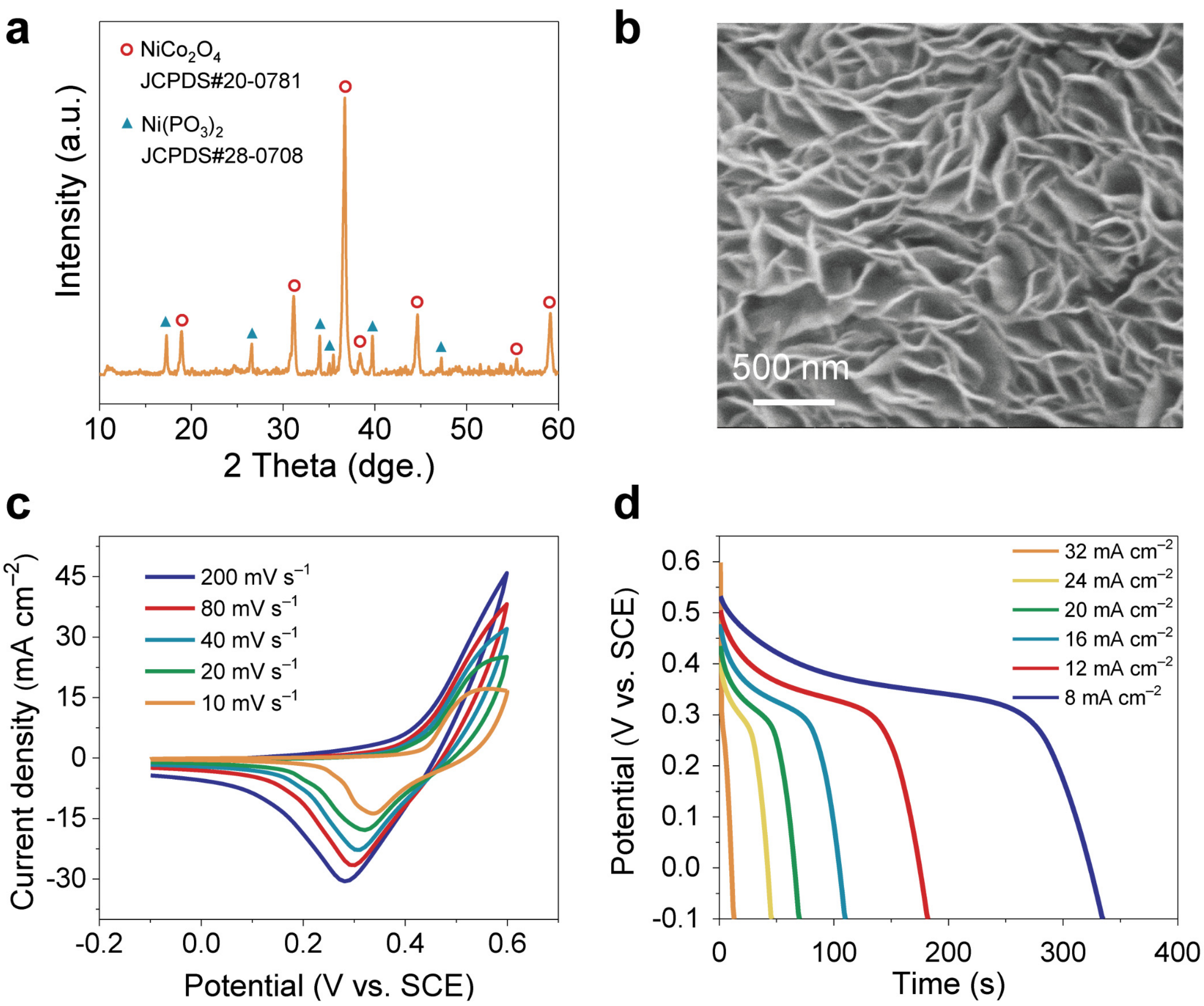

Supplementary Figure 22 **a** XRD spectrum, **b** SEM image, **c** CV curves under different scan rates and **d** GCD curves under different current densities of P-$NiCo_2O_4$ electrode.

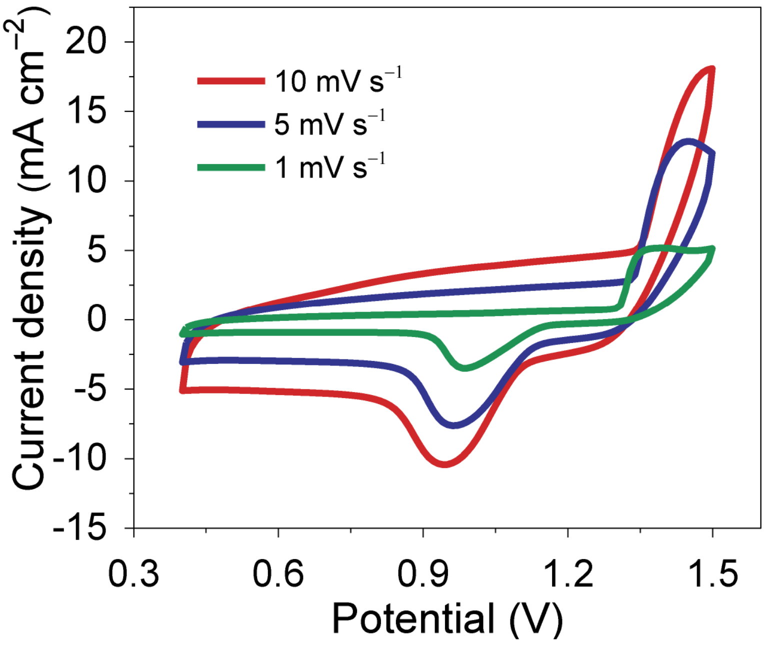

Supplementary Figure 23 CV curves under different scan rates of the NiCo//Sb battery.

Supplementary Notes

Supplementary Note 1 Working mechanism on the NiCo//Sb battery

Cathode reaction:

$NiCo_2O_4 + OH^- + H_2O \rightleftharpoons NiOOH + 2CoOOH + e^-$ Supplementary Equation (1)

$CoOOH + OH^- \rightleftharpoons CoO_2 + H_2O + e^-$ Supplementary Equation (2)

Anode reaction:

${SbO_2}^- + H_2O + 3e^- \rightleftharpoons Sb + 4OH^-$ Supplementary Equation (3)

Full cell reaction:

$NiCo_2O_4 + {SbO_2}^- + H_2O \rightleftharpoons NiOOH + 2CoO_2 + Sb + OH^-$ Supplementary Equation (4)

Supplementary Note 2 Reaction equation for the formation of SbO_2^-

The φ-pH diagram for H_2O-Sb and experimental results reported in previous works (refs. S1-S3) have fully demonstrated that the Sb (III) exists as SbO_2^- in alkaline solution. The reaction equation for the formation of SbO_2^- is listed below:

$$Sb^{3+} + 4OH^- \rightarrow SbO_2^- + 2H_2O$$ Supplementary Equation (5)

Supplementary Note 3 Calculations about capacity, energy density and power density of Sb anode and NiCo//Sb battery

According to the galvanostatic charge/ discharge curves, the areal capacities of the Sb deposition electrodes on CS or FCS is calculated and the equation is as follows:

$$C_s = \frac{\int_0^{\Delta t} I \times dt}{S} \quad \text{Supplementary Equation (6)}$$

where C_s (mA h cm^{-2}) is the areal capacity, Δt (h) is the discharging time, I (mA) is the constant discharging current and S (cm^2) is the area of Sb deposition electrode.

The areal capacity of the NiCo//Sb battery is calculated similarly to that of a single electrode by the following equation:

$$C_{cell} = \frac{\int_0^{\Delta t} I \times dt}{S} \quad \text{Supplementary Equation (7)}$$

where C_{cell} (mA h cm^{-2}) is the areal capacity of the NiCo//Sb battery, Δt (h) is the discharging time, I (mA) is the constant discharging current and S (cm^2) is the area of the NiCo//Sb battery.

The energy density (E) and specific power density (P) of the NiCo//Sb battery are obtained from the following equations:

$$E = \frac{\int_{V_1}^{V_2} C_{cell} \times dV}{d} \quad \text{Supplementary Equation (8)}$$

$$P = \frac{E}{1000 \times \Delta t} \quad \text{Supplementary Equation (9)}$$

where E (mW h cm^{-3}) is the energy density of the NiCo//Sb battery, V_1 (V) and V_2 (V) are the starting and ending voltages of the voltage window, C_{cell} is the areal capacities obtained from Supplementary Equation (7), V (V) is the voltage window and d is the combined thickness of the cathode and the anode (0.08 cm). P (W cm^{-3}) is the specific power density of the NiCo//Sb battery and Δt (h) is the discharging time.

Supplementary References

1. Ulrich, N. Speciation of antimony(III), antimony(V) and trimethylstiboxide by ion chromatography with inductively coupled plasma atomic emission spectrometric and mass spectrometric detection. *Anal. Chim. Acta* **359**, 245-253 (1998).
2. Vink, B. W. Stability relations of antimony and arsenic compounds in the light of revised and extended Eh-pH diagrams. *Chem. Geol.* **130**, 21-30 (1996).
3. Wang, Q. & Wang, Y. Fundamental electrochemical behavior of antimony in alkaline solution. *Journal of Sustainable Metallurgy* **5**, 606-616 (2019).
